# MicroRNA-320a enhances LRWD1 expression through the AGO2/FXR1-dependent pathway to affect cell behaviors and the oxidative stress response in human testicular embryonic carcinoma cells

**DOI:** 10.18632/aging.205571

**Published:** 2024-02-21

**Authors:** Yi-Hsiung Lin, Chia-Hui Su, Hsin-Mei Chen, Ming-Syuan Wu, Hsien-An Pan, Chia-Ning Chang, Yu-Sheng Cheng, Wen-Tsan Chang, Chien-Chih Chiu, Yen-Ni Teng

**Affiliations:** 1 Center for Lipid Biosciences, Department of Medical Research, Kaohsiung Medical University Hospital, Kaohsiung Medical University, Kaohsiung 807, Taiwan; 2 Division of Cardiology, Department of Internal Medicine, Kaohsiung Medical University Hospital, Kaohsiung Medical University, Kaohsiung 807, Taiwan; 3 Regenerative Medicine and Cell Therapy Research Center, Kaohsiung Medical University, Kaohsiung 807, Taiwan; 4 Department of Biological Sciences and Technology, National University of Tainan, Tainan 700, Taiwan; 5 An-An Women and Children Clinic, Tainan 704, Taiwan; 6 Department of Urology, National Cheng Kung University Hospital, College of Medicine, National Cheng Kung University, Tainan 701, Taiwan; 7 Department of Surgery, Division of General and Digestive Surgery, Kaohsiung Medical University Hospital, Kaohsiung 807, Taiwan; 8 Department of Biotechnology, Kaohsiung Medical University, Kaohsiung 807, Taiwan; 9 Department of Biological Sciences, National Sun Yat-Sen University, Kaohsiung 804, Taiwan

**Keywords:** testicular cancer, LRWD1, miR-320a, AGO2, FXR1

## Abstract

Background: Testicular cancer is fairly rare but can affect fertility in adult males. Leucine-rich repeats- and WD repeat domain-containing protein 1 (LRWD1) is a sperm-specific marker that mainly affects sperm motility in reproduction. Our previous study demonstrated the impact of LRWD1 on testicular cancer development; however, the underlying mechanisms remain unclear.

Methods: In this study, various plasmids associated with LRWD1 and miR-320a manipulation were used to explore the roles and regulatory effects of these molecules in NT2D1 cellular processes. A Dual-Glo luciferin-luciferase system was used to investigate LRWD1 transcriptional activity, and qRT-PCR and western blotting were used to determine gene and protein expression.

Results: The results suggested that miR-320a positively regulated LRWD1 and positively correlated with NT2D1 cell proliferation but negatively correlated with cell migration and invasion ability. In addition, the miRNA-ribonucleoprotein complex AGO2/FXR1 was shown to be essential in the mechanism by which miR-320a regulates LRWD1 mRNA expression. As miR-320a was required to regulate LRWD1 expression through the AGO2 and FXR1 complex, eEF2 and eLF4E were also found to be involved in miR-320a increasing LRWD1 expression. Furthermore, miR-320a and LRWD1 were responsive to oxidative stress, and NRF2 was affected by the presence of miR-320a in response to ROS stimulation.

Conclusions: This is the first study showing the role of miR-320a in upregulating the testicular cancer-specific regulator LRWD1 and the importance of the AGO2/FXR1 complex in miR-320a-mediated upregulation of LRWD1 during testicular cancer progression.

## INTRODUCTION

Testicular cancer occurs in the testicles, which are in the scrotum, the loose sac of skin under the penis. Although testicular cancer is rare, it is the most common cancer among American men between the ages of 20 and 40. Even when it has spread beyond the testicles, this type of cancer has a high rate of successful treatment [1].

Leucine-rich repeats and WD repeat domain-containing protein 1 (LRWD1) is a testis-specific protein that is mainly expressed in the centrosome of the neck of sperm. Microarray analysis was used to identify LRWD1 as a crucial regulator related to spermatogenesis [2]. In 2010, LRWD1 was first reported as a candidate centrosomal protein and was highly associated with spermatozoa maturation [3]. It was found to colocalize and interact with γ-tubulin, which is responsible for microtubule organization. Studies using chromatin immunoprecipitation (ChIP) analysis and gel electrophoretic mobility shift assay (EMSA) identified the core promoter region and main interacting factors of LRWD1 that regulate its biological function, including NF-κB. The results showed that NF-κB is an important regulator of the expression of LRWD1, and this regulation of downstream genes relies on the activation and transcription initiation of the conserved κB site in the prom r region of LRWD1 [4]. In addition, there is evidence that LRWD1 is involved in DNA replication and mainly associates with chromatin during the G1 phase of the cell cycle. The direct association of LRWD1 and histone repressive marks (H3K9 methylation) suggests a role for LRWD1 in recruiting the H3K9me3 mark to certain genomic loci [5]. Recently, we reported that autophagy is activated in LRWD1-deficient testicular cancer cells. When cells lacked LRWD1, autophagy inhibition via chloroquine or bafilomycin A1 promoted the death of testicular cancer cells. These findings confirmed that LRWD1 controls microtubule nucleation and cell cycle progression in human embryonic testicular cancer cells and revealed its role in autophagy regulation [2].

MicroRNAs (miRNAs) are a type of ribonucleic acid with a length of approximately 20 to 24 nucleotides that are widely found in eukaryotes. It can affect the stability of mRNA and regulate gene performance by binding to the 3′-UTR. These RNAs are derived from the primary transcript, that is, pri-miRNA, which is transformed into the stem loop of pre-miRNA. The length of pri-miRNAs is approximately 300~1000 bases [6]. After the first step in the process, pri-miRNA becomes pre-miRNA, that is, a miRNA precursor, with a length of approximately 70~90 fragments, after which it finally becomes a functionally mature miRNA. After the pre-miRNA is digested by Dicer, it becomes a mature miRNA. The mature miRNA is inserted into the RNA-induced silencing complex (RISC) to recognize the target mRNA and binds to cause translational inhibition or constant binding of the target mRNA [7]. miR-320a is a novel miRNA that was first reported in 2017 as a suppressor of gliomas. Mechanistically, by directly targeting SND1 and β-catenin, miR-320a was found to increase the tumor suppressor p21 WAF1 and decrease Smad2, Smad4, MMP2, MMP7 and cyclin D1 [8]. In addition, miR-320a is reported to be involved in the biological function of metastasis-associated lung adenocarcinoma transcript 1 (MALAT1), as MALAT1 knockdown induced upregulation of miR-320a, which directly bound to FOXM1 and repressed FOXM1 expression in HUVECs [9].

There are four types of AGO proteins in mammals: AGO1, AGO2, AGO3 and AGO4. AGOs combine with miRNA or siRNA to form an RISC to inhibit the translation of mRNA or promote its degradation. Past studies have revealed that AGO2 plays a very important role in mammalian development, survival and resistance to viral infections. Double-stranded miRNA molecules quickly combine with proteins, including AGO, to form an RISC, also known as miRNP (microribonucleoprotein), which inhibits gene expression. [10]. The RISC can adjust the effect of the AGO protein in response to specific cell conditions, such as HuR-mediated reduction of transcription inhibition [11] or the stimulatory effect of AGO2/FXR1 on translation [12]. miR-122 was also reported to be involved in the AGO2-associated stimulatory effect on RNA replication of hepatitis C virus [13]. AGO2, which contains four functional core domains, the N domain, PAZ domain, MID domain, and PIWI domain from the N-terminus to the C-terminus, is the only member with catalytic activity in the Argonaute family and the core component of miRNP [14, 15]. The PAZ and PIWI domains bind the 3′ and 5′ terminal nucleotides, respectively, anchoring the single-stranded miRNA into the groove [16–18]. AGO2 was found to interact with miR-122 and enhance HCV RNA amplification. AGO2 deletion resulted in the repression of HCV RNA amplification in a transient HCV replication assay, and AGO2 knockdown caused a reduction in HCV translation. These results show the importance of AGO2 in miRNA-initiated gene accumulation and translation [19]. AGO2 was recently reported to have a biological function in recruiting other effectors of translational repression/mRNA destabilization, mainly through the phosphorylation of LIM domain-containing proteins. In the absence of LIMD1 and AGO2, the miRNA silencing mechanism switches to interactions with AGO3 and WTIP [20].

Our previous findings have demonstrated that reduced levels of LRWD1 in cells are associated with reduced cell growth and mitosis and affect cellular microtubule atrophy rates [2]. Since DNA methylation plays an important regulatory role in gene expression, it is also one of the potential mechanisms affecting LRWD1 expression [21]. Previous studies have demonstrated the impact of LRWD1 on testicular cancer development; however, the underlying mechanisms remain unknown. We found that miRNAs can regulate the expression of LRWD1, and the mechanism involved is worth discussing. Here, we report the role of miR-320a in the transcriptional regulation of LRWD1. A Dual-Glo luciferin-luciferase system was used to assess LRWD1 transcriptional activity, and qRT-PCR and western blotting were used to determine gene and protein expression in NT2D1 cells. Furthermore, the importance of the miRNA-ribonucleoprotein complex AGO2/FXR1 in the mechanism by which miR-320a regulates LRWD1 mRNA expression was proven. This study supports the targeting of LRWD1 or miR-320a to reduce testicular cancer progression.

## RESULTS

### miR-320a positively regulated the expression of LRWD1 by targeting its 3′UTR

**Figure 1:**
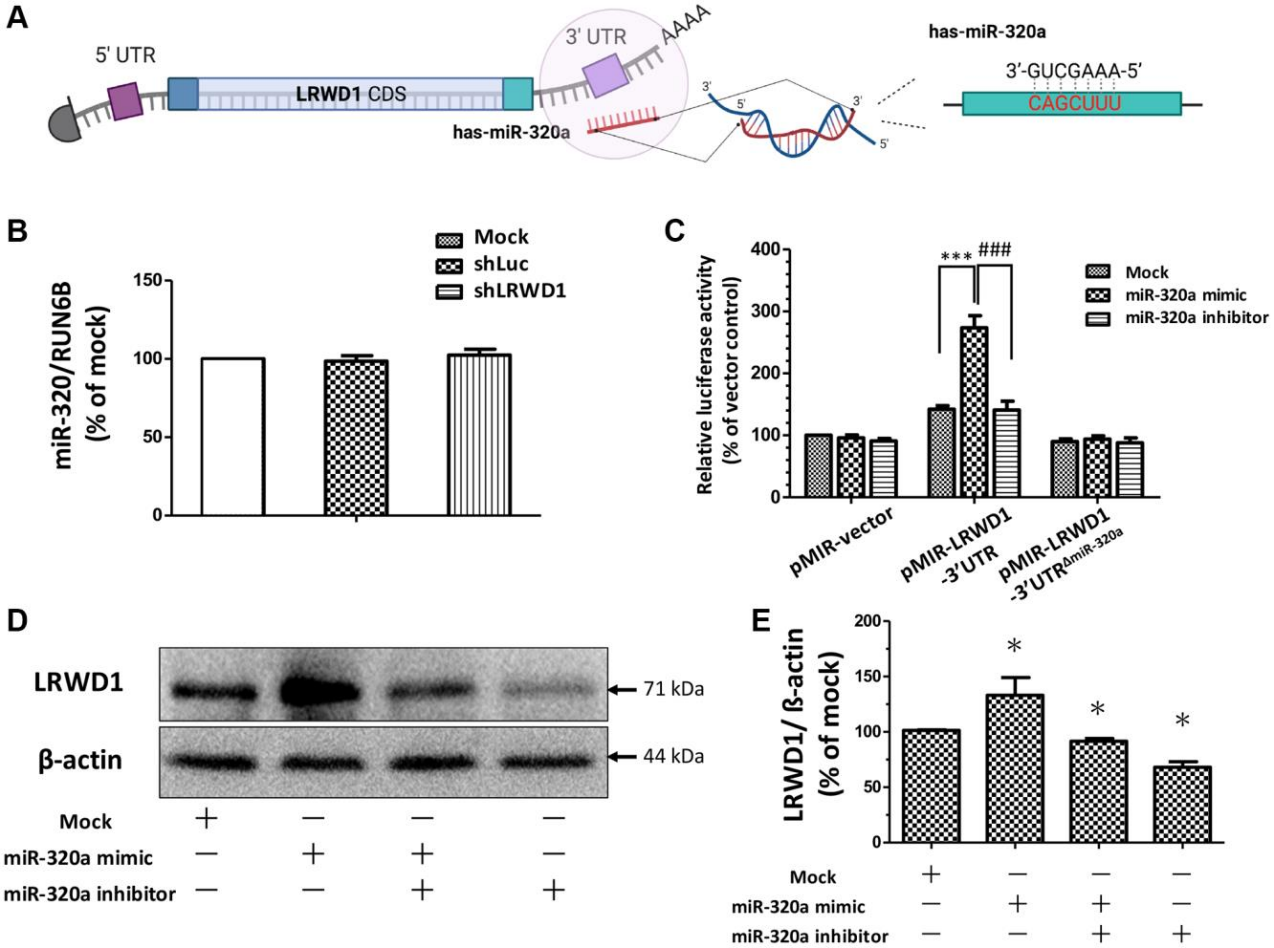
**The effect of miR-320a in regulating LRWD1 expression.** (**A**) The predicted binding sequence of miR-320a on the LRWD1 3′UTR. (**B**) The effect of the deletion of LRWD1 on regulating endogenous miR-320a expression. (**C**) The regulatory effect of miR-320a on LRWD1 expression was demonstrated using transfecting cells with the miR-320a mimic or inhibitor by luciferase assay. (**D**) The effects of the miR-320a mimic and inhibitor on protein expression. (**E**) Quantitative analysis of LRWD1 protein expression regulated by the miR-320a mimic and inhibitor.

To determine the regulatory relationship between miR-320a and LRWD1, we first performed a BLAST search for miR-320a binding sites in the whole mRNA sequence of LRWD1. The schematic diagram shows the binding site of miR-320a in the LRWD1 3′UTR (Figure 1A). To further determine the regulatory effect of miR-320a on LRWD1, specific shRNAs, miR-320a inhibitors and mimics were used. shLRWD1 was used to silence LRWD1 mRNA expression, and we found that compared with that in the control groups (mock and shLuc), miR-320a expression in the shLRWD1 group was not affected, while the expression of LRWD1 was knocked down (Figure 1B). Conversely, by using the miR-320a mimic to increase the relative miR-320a activity, we found that the miR-320a mimic dramatically increased the relative luciferase activity in NT2D1 cells transfected with the LRWD1 3′UTR plasmid (pmiR-LRWD1-3′UTR). Further evidence was provided by cotransfection of cells with the miR-320a mimic and an LRWD1-3′UTR plasmid with a deleted miR-320a binding sequence (pmiR-LRWD1-3′UTR^ΔmiR-320a^). The miR-320a mimic failed to increase the luciferase activity in the pmiR-LRWD1-3′UTR^ΔmiR-320a^ group when the miR-320a binding region was deleted (Figure 1C). In addition, the miR-320a inhibitor showed no influence on the luciferase activity of either the pmiR-LRWD1-3′UTR or pmiR-LRWD1-3′UTRΔmiR-320a plasmid compared with that of control pMIR-REPORT plasmid-transfected cells (Figure 1C). The miR-320a mimic enhanced LRWD1 expression, and this effect was significantly reduced by the miR-320a inhibitor (Figure 1D, 1E). These results suggest that miR-320a exerted a regulatory effect on LRWD1 by targeting the 3′UTR and positively regulated the protein expression of LRWD1 in NT2D1 cells.

### miR-320a in regulating cell behaviors in NT2D1 cells

**Figure 2:**
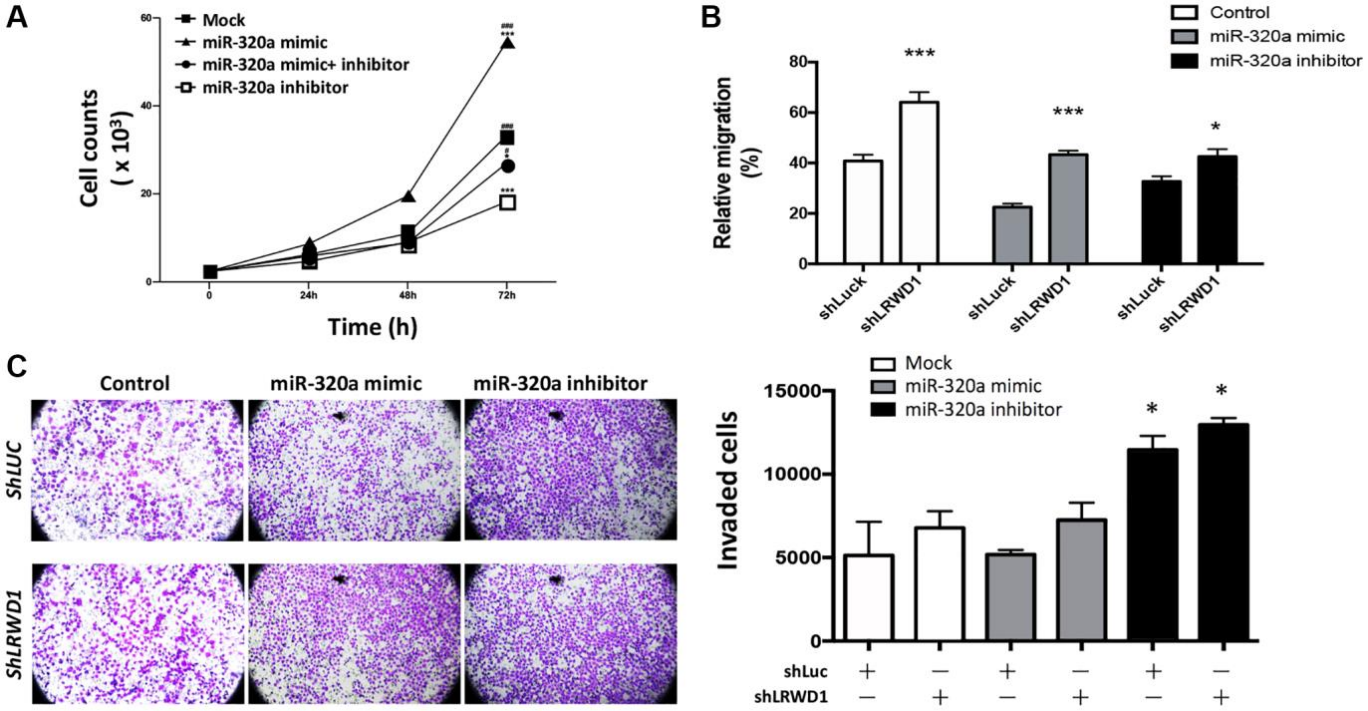
**Regulatory effect of miR-320a on NT2D1 cell behaviors.** (**A**) NT2D1 viability was affected by the miR-320a mimic and inhibitor. The treatment groups included the mock, miR-320a mimic, and miR-320a inhibitor groups. (**B**) Cell migration regulated by the miR-320a mimic/inhibitor and shLRWD1 in the presence or absence of LRWD1 gene expression. (**C**) The effect of the miR-320a mimic/inhibitor and shLRWD1 in regulating NT2D1 cell invasion ability in the presence or absence of LRWD1 gene expression.

Our previous study showed that LRWD1 was positively associated with cell proliferation; thus, the effect of miR-320a on cell behaviors, including proliferation, migration and invasion, was investigated. The MTT assay showed that miR-320a mimic-treated NT2D1 cells exhibited increased cell proliferation activity compared with the control mock-treated cells, and this phenomenon was decreased by the miR-320a inhibitor, suggesting that miR-320a positively regulated cell proliferation (Figure 2A).

Cell migration was examined by using a wound-healing assay. Figure 2B shows that compared to the migration of control NT2D1 cells, there was a slight decrease in the migration capability of miR-320a mimic-transfected NT2D1 cells and a great improvement in the migration of miR-320a inhibitor-transfected NT2D1 cells. In addition, compared with shLuc-transfected NT2D1 cells, LRWD1-knockdown cells exhibited significantly increased cell migration with or without the miR-320a mimic or inhibitor. The results suggest that miR-320a and LRWD1 negatively regulate NT2D1 cell migration (Figure 2B).

We next investigated the effect of miR-320a on cell invasion. NT2D1 cells lacking LRWD1 exhibited enhanced cell invasion, and transfection of the miR-320a mimic increased the cell invasion ability even in the absence of LRWD1 expression. Conversely, the miR-320a inhibitor improved LRWD1 deficiency-mediated enhancement of cell invasion (Figure 2C). The results suggest that miR-320a negatively regulates the migration and invasion of NT2D1 cells.

### Knockdown of AGO2 and FXR1 correlated with a reduction in the miR-320a-mediated upregulation of LRWD1

**Figure 3:**
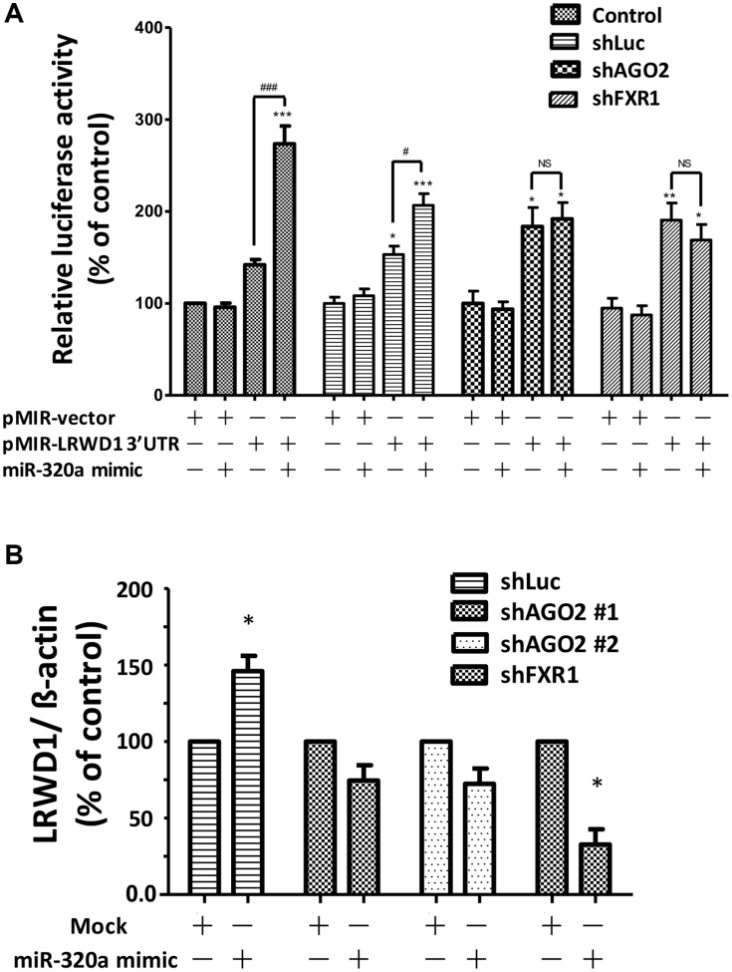
**Knockdown of AGO2 and FXR1 was correlated with a reduction in miR-320a-mediated upregulation of LRWD1.** The effect of the presence and absence of AGO2 and FXR1 expression in regulating (**A**) LRWD1 expression by luciferase assay. (**B**) LRWD1 gene expression by qRT-PCR using AGO2- and FXR1-specific shRNAs (shLuc: negative control; shAGO2: AGO2 shRNA; shFXR1: FXR1 shRNA; ^*^p < 0.05, ^**^p < 0.01, ^***^p < 0.001 compared with the vector-only control group; Abbreviation: NS, no significance).

We further explored the underlying mechanism associated with the regulation of LRWD1 by miR-320a. Previous reports indicated that the transcription factors AGO2 and FXR1 are likely to be involved in the observed regulatory effect. Therefore, shRNAs targeting AGO2 and FXR1 were used to explore the mechanism by which miR-320a regulates LRWD1 gene expression. Different plasmids, namely, vector only, miR-320a binding sequence, LRWD1 3′UTR, and LRWD1 3′UTR+miR-320a binding sequence, were cotransfected for LRWD1 luciferase activity measurement. We found that in the mock and shLuc groups, the luciferase activity of the LRWD1 3′UTR+miR-320a binding sequence plasmid was increased, proving the activation of LRWD1. However, both shAGO2 and shFXR1 transfection significantly reduced the luciferase activity of the LRWD1 3′UTR+miR-320a binding sequence plasmid (Figure 3A). In addition, qRT-PCR was used to quantify LRWD1 gene expression. The miR-320a mimic enhanced the gene expression of LRWD1, and this enhancement was reversed by shAGO2 and shFXR1 (Figure 3B). These results indicate that miR-320a regulates the gene expression of LRWD1 through the transcription factors AGO2 and FXR1.

### Overexpression of AGO2 and FXR1 enhanced the miR-320a-initiated increase in LRWD1 gene expression

**Figure 4:**
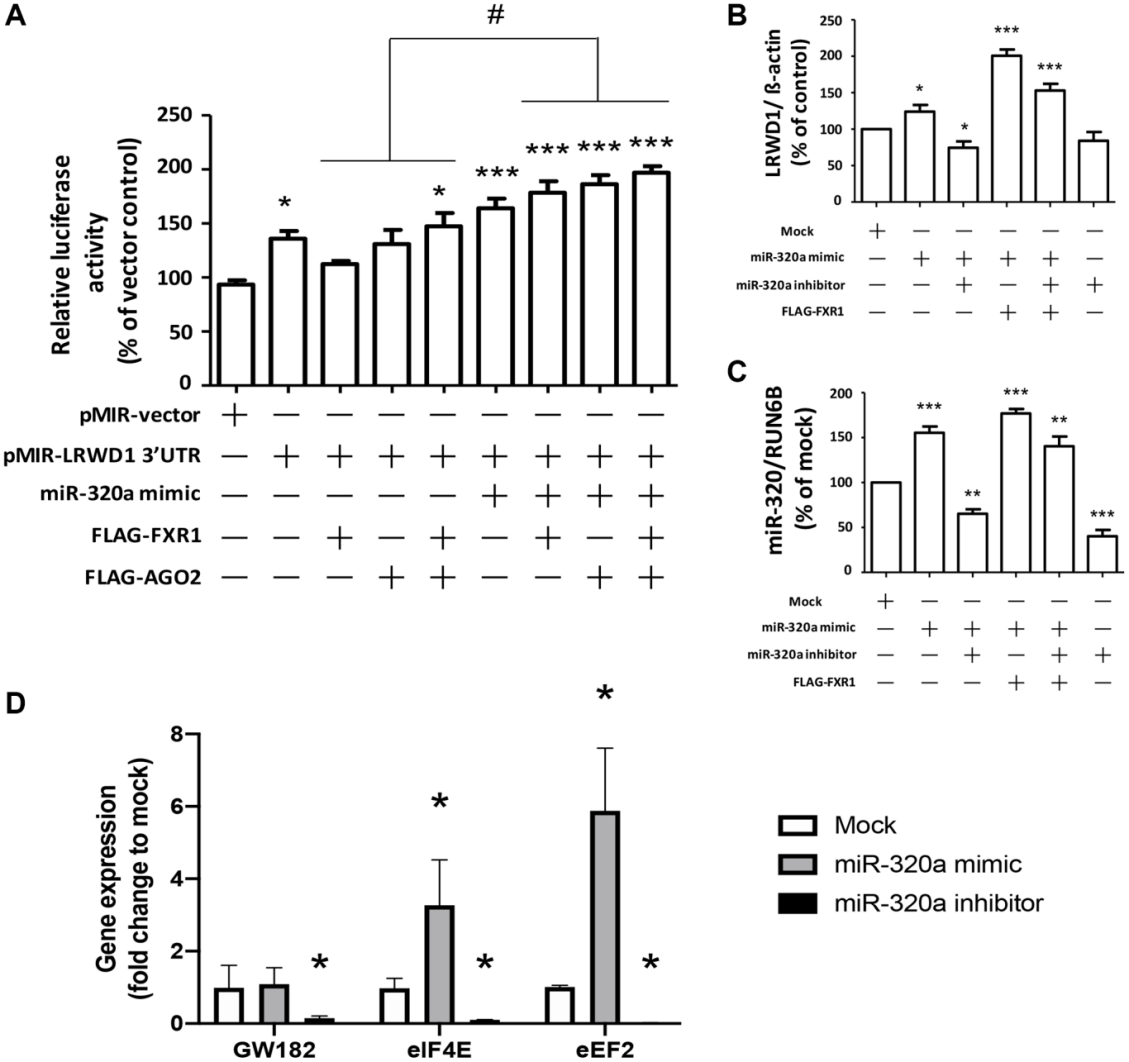
**Overexpression of AGO2 and FXR1 enhanced the miR-320a-mediated increase in LRWD1 gene expression.** (**A**) The enhancing effect of AGO2 and FXR1 overexpression in promoting LRWD1 expression was investigated by using the pMIR-LRWD1 3′UTR plasmid, miR-320a mimic and FXR1/AGO2 overexpression FLAG-tag plasmid. qRT-PCR results of LRWD1 gene expression regulated by overexpression of (**B**) FXR1 and (**C**) AGO2 under treatment with the miR-320a mimic or inhibitor. (**D**) The potential AGO2 and binding elements gene expression which directly increased miRNA target gene transcription. (^*^p < 0.05, ^**^p < 0.01, ^***^p < 0.001 compared with the vector control or negative control; ^###^*p* < 0.001 compared with the miR-320a mimic-treated group).

We next investigated the effect of AGO2 and FXR1 overexpression on miR-320a-induced LRWD1 gene upregulation. The miR-320a mimic and LRWD1 3′UTR plasmids were cotransfected into cells overexpressing AGO2 and FXR1 to investigate their effects on luciferase activity. We found that overexpression of AGO2 or FXR1 alone induced only a moderate increase in LRWD1 3′UTR luciferase activity, whereas overexpression of both induced a significant increase. The additional treatment with miR-320a mimic combined with overexpression of AGO2 or FXR1 further enhanced the LRWD1 3′UTR luciferase activity, and overexpression of both AGO2 and FXR1 plus treatment with miR-320a mimic resulted in the greatest enhancement of luciferase activity (Figure 4A). We also used qRT-PCR to quantify LRWD1 gene expression and found that overexpression of FXR1 enhanced the miR-320a mimic-mediated increase in LRWD1 gene expression and reversed the suppressive effect of the miR-320a inhibitor on LRWD1 expression (Figure 4B). Similarly, miR-320a mimic and inhibitor treatment was combined with the same FXR1 overexpression to monitor the expression of miR-320a. We found that FXR1 overexpression combined with miR-320a mimic treatment only slightly increased the miR-320a expression level; however, there was no significant difference compared with the expression after treatment with the miR-320a mimic alone. In addition, the addition of the miR-320a inhibitor only slightly reduced the expression of miR-320a, and there was no significant difference from the level after treatment with the miR-320a mimic plus FXR1 overexpression (Figure 4C). These results indicate that AGO2 and FXR1 have a positive regulatory effect on LRWD1 but exhibit only a slight regulatory effect on miR-320a expression. In addition, we also conducted experiments on potential factors that promote miRNA-320a to enhance the expression of the target LRWD1 gene. We found that the expression of eIF4E and eEF2, which could enhance target gene expression, was also enhanced under treatment with the miRNA-320a mimic. Similarly, these genes were also downregulated after miR-320a inhibitor treatment (Figure 4D). However, GW182 did not undergo significant changes under miRNA-320a mimic treatment but was downregulated by miR-320a inhibitor treatment.

### Overexpression of AGO2 and FXR1 enhanced the miR-320a-mediated increase in LRWD1 protein expression

**Figure 5:**
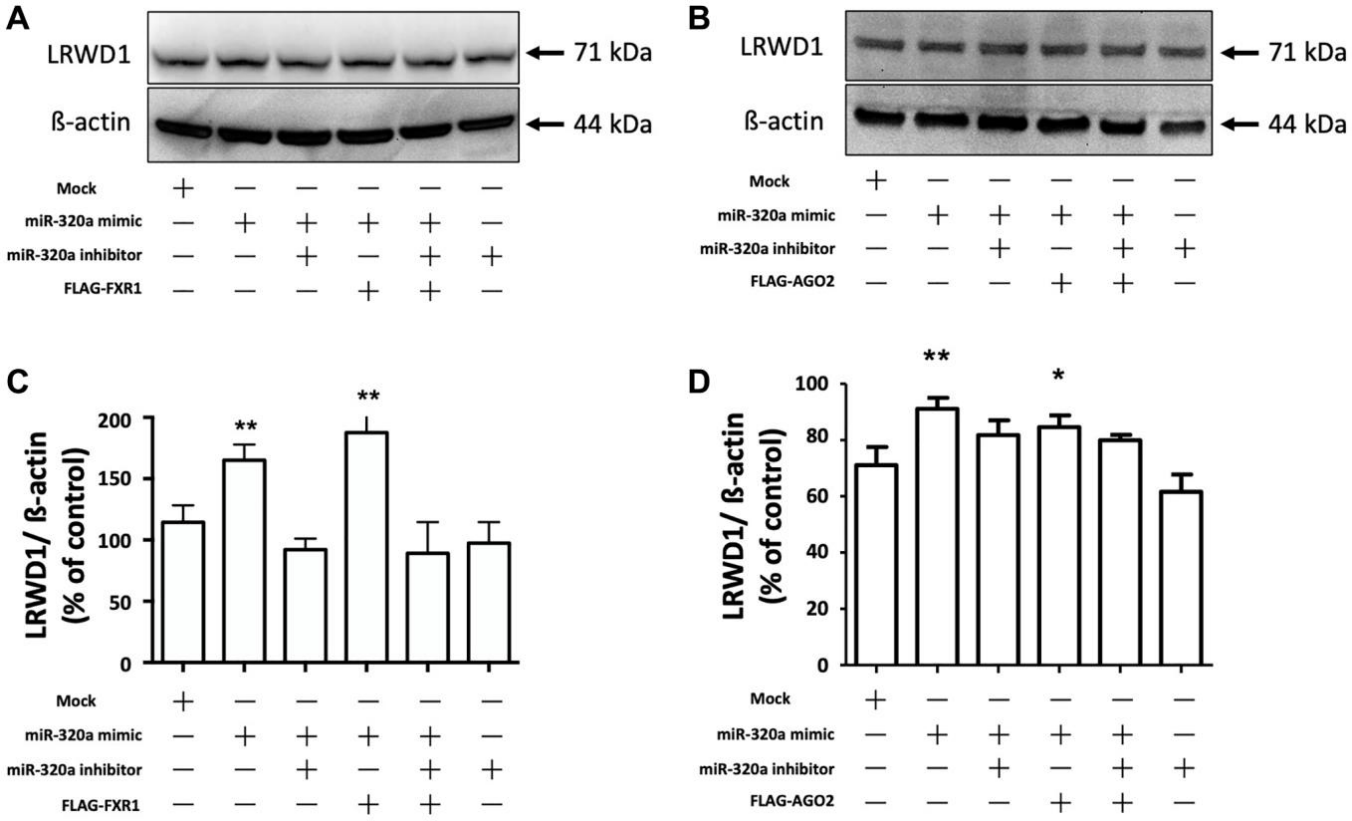
**Overexpression of AGO2 and FXR1 enhanced the miR-320a-mediated increase in LRWD1 protein expression.** Western blotting was performed to determine the protein expression of LRWD1 in cells overexpressing (**A**) FXR1 and (**B**) AGO2 in the presence or absence of the miR-320a mimic and inhibitor. Quantitative analysis of LRWD1 protein expression modified by (**C**) FXR1 and (**D**) AGO2 overexpression showed the significance of the regulatory effect of the miR-320a mimic and inhibitor (^*^p < 0.05, ^**^p < 0.01, ^***^p < 0.001 compared with the negative control).

We next determined the effect of AGO2 and FXR1 on the miR-320a-mediated regulation of LRWD1 protein expression. The results showed that the miR-320a mimic increased the protein expression of LRWD1, and the miR-320a inhibitor inhibited the protein expression of LRWD1. Overexpression of FXR1 further significantly enhanced the effect of the miR-320a mimic on LRWD1, but it did not reverse the effect of the miR-320a inhibitor in reducing LRWD1 protein expression (Figure 5A, 5B). Similarly, AGO2 overexpression increased the effect of the miR-320a mimic on LRWD1 protein expression and failed to restore LRWD1 protein expression reduced by the miR-320a inhibitor (Figure 5C, 5D). These findings indicate that the mechanism related to modifying protein expression was different from that seen at the genetic level.

### miR-320a, LRWD1 and cellular antioxidant capacity

**Figure 6:**
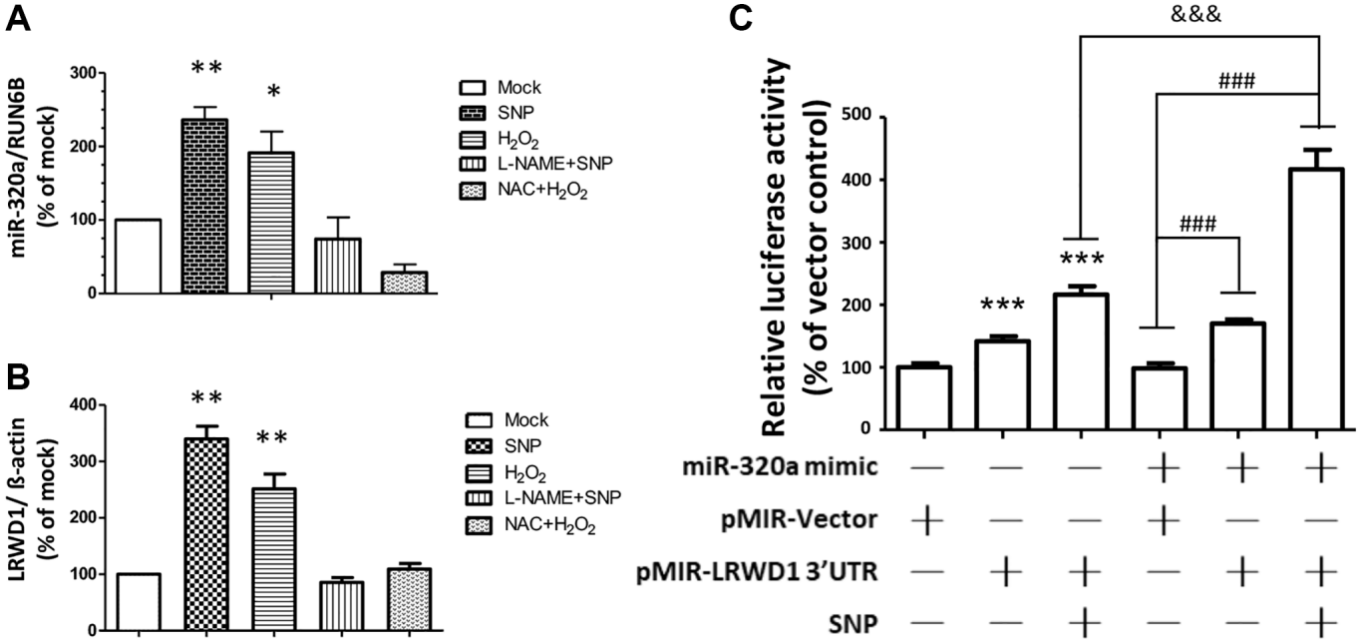
**LRWD1 and miR-320a expression and their regulatory effect in response to oxidative stress.** The gene expression of (**A**) miR-320a and (**B**) LRWD1 responding to the oxidative stress OH^-^ (H_2_O_2_), NO donor (SNP), and the individual inhibitor treatments. (**C**) The activity of LRWD1 stimulated by miR-320 mimic and SNP in NT2D1 cells (^***^p < 0.001 compared with the vector-only control; ^###^*p* < 0.001 and ^&&&^*p* < 0.001 compared with the indicated groups).

According to previous research results, LRWD1 is involved in cellular antioxidant mechanisms. Therefore, we also determined the regulatory effect of miR-320a and LRWD1 in response to cellular oxidative stress. As shown in Figure 6A, 6B, NT2D1 cells were treated with sodium nitroprusside (SNP, a nitric oxide (NO) donor) and hydrogen peroxide (H_2_O_2_), and the gene expression of LRWD1 and miR-320a was significantly increased.

The LRWD1 and miR-320a genes were expressed normally after oxidative stress was eliminated by adding the individual inhibitors L-NAME and NAC to remove free radicals, and there was no significant difference between the control and individual inhibitor treatment groups (Figure 6A, 6B).

We further verified the regulatory effect of miR-320a on LRWD1 under oxidative stress by using miR-320a mimic and LRWD1 promoter assay plasmids. We found that in the group in which SNPs and LRWD1 reporter plasmids were added, the activity of LRWD1 was significantly increased by more than 2-fold, and in the group in which miR-320a mimic and LRWD1 reporter plasmids were added, LRWD1 expression was also increased by nearly 2-fold. Interestingly, after adding the miR-320a mimic, LRWD1 reporter plasmid and SNP treatment, we found that the activity of LRWD1 was greatly increased by more than 4-fold (Figure 6C). The results indicate that increasing miR-320a in NT2D1 cells could significantly improve the expression and activity of LRWD1 in response to cellular oxidative stress.

### NRF2 is involved in miR-320a-regulated LRWD1 expression and subsequent antioxidant capacity

**Figure 7:**
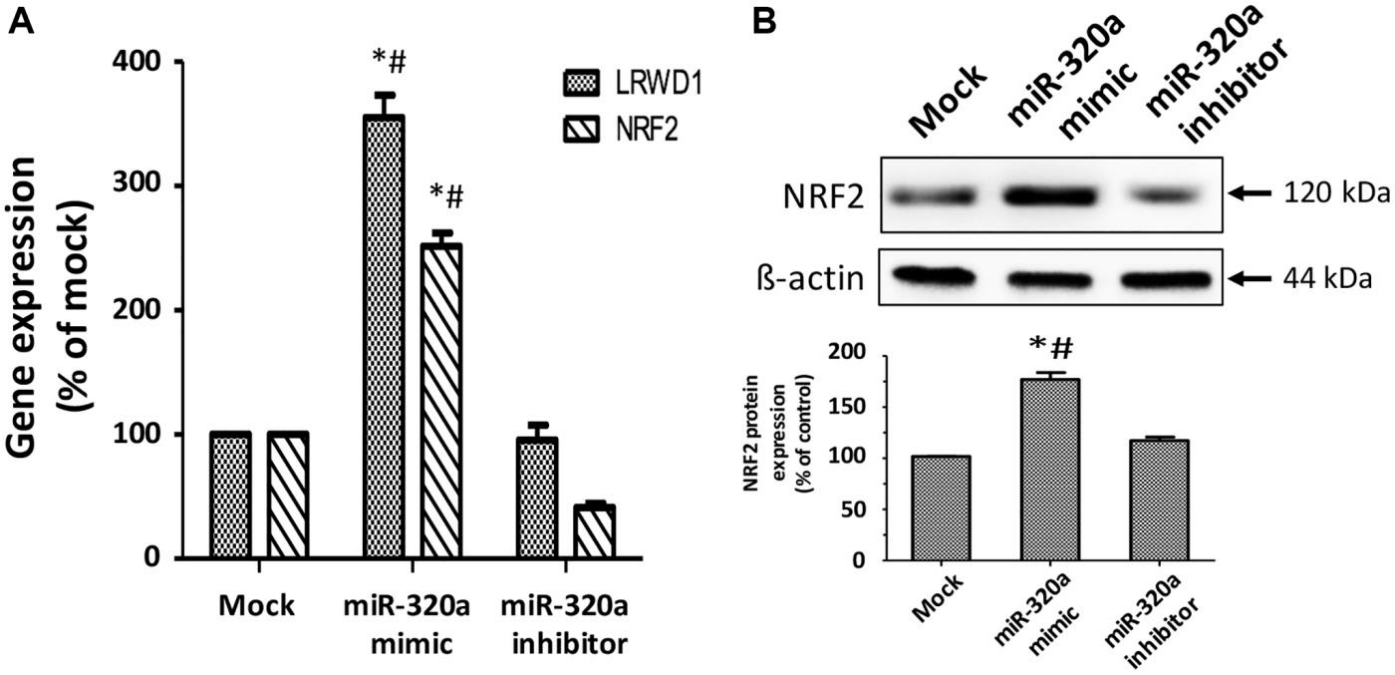
**Regulation of miR-320a in the LRWD1 and NRF2 genes and NRF2 protein expression.** (**A**) qRT-PCR results reveal LRWD1 and NRF2 gene expression under stimulation with miR-320a mimic and inhibitor. (**B**) Western blot images and quantitative results (bottom panel) of NRF2 protein regulated by miR-320a mimic and inhibitor. ^*^p < 0.05 compared with individual mock controls, and ^#^*p* < 0.05 compared with individual miR-320a inhibitors.

Our previous study also confirmed that NRF2 was activated to enhance the expression and activity of LRWD1 to protect cells against oxidative stress. In the current study, we investigated the regulatory effect between miR-320a and NRF2 during cellular oxidative stress. We found that NT2D1 containing miR-320a mimics increased the gene expression of LRWD1 (3.6-fold) and NRF2 (2.4-fold). When the miR-320a inhibitor was present, the gene expression of LRWD1 was normal, and NRF2 was further reduced (Figure 7A). Similarly, the miR-320a mimics improved the protein expression of NRF2, and the miR-320a inhibitor maintained its protein expression at a normal level (Figure 7B). The results support that miR-320a positively regulates the expression of LRWD1 and NRF2.

## DISCUSSION

### General discussion

Our previous report showed that LRWD1 plays an important role in maintaining the survival of human testicular embryonic cancer cells and promotes cell proliferation and that its related mechanisms are worthy of further study. However, the underlying mechanism regarding the role of LRWD1 in carcinoma cells remains unclear. In the current study, we aimed to reveal more information on the biological function of LRWD1 in regulating cell function and its regulatory effects on carcinoma cells. Our results showed that LRWD1 affected not only cell proliferation but also cell migration and invasion. However, the regulatory effects on migration and invasion were negative and different from those on cell proliferation. In addition, we identified a miRNA, miR-320a, that exerted a positive regulatory effect on LRWD1, unlike most other miRNAs, which negatively regulate gene expression. The essential component proteins of miRNA biogenesis, AGO2 and FXR1, were also proven to be greatly involved in the regulatory effects of miR-320a on LRWD1 gene transcription and downstream cell behaviors.

### LRWD1 regulated cell behaviors

The effect of LRWD1 in promoting NT2D1 cell proliferation was revealed by our previous study; however, its effect on other cell behaviors, such as migration and invasion, remained unclear. LRWD1 is highly involved in cell cycle progression, and a lack of LRWD1 results in a comprehensive reduction in cyclin E, A, and CDK2 phosphorylation [2]. In the current study, we provide evidence suggesting that LRWD1 negatively regulates cell migration and invasion. The cell migration and Transwell invasion results indicated that knocking down LRWD1 with a specific shRNA in NT2D1 cells increased the capability of the cells to migrate into the central area of the scratch and penetrate through the Transwell membrane. The data clearly support the role of LRWD1 in the negative regulation of NT2D1 cell migration and invasion. Most of the references indicate that LRWD1 is highly involved in maintaining telomere activity and the associated mechanism by targeting ALT telomeres to control homologous recombination (HR)-based mechanisms [22, 23]. The binding of LRWD1 with two regulators, RPA and RAD51, could also affect the tightening of ssDNA and chromatin histones [23, 24]. However, this evidence only supports that LRWD1 is closely related to cell growth but not migration or invasion. This is the first time that the role of LRWD1 in cell behaviors has been revealed during testicular cancer development, and a miRNA, miR-320a, was found to regulate the expression and activity of LWRD1 and subsequent cell behaviors. The miR-320a mimic improved the expression of LRWD1 and enhanced its effect on cell behaviors; in contrast, the miR-320a inhibitor reversed these effects. We believe that the positive regulation of miRNAs and specific molecules in testicular cancer cells is rare and worthy of further investigation.

### miR-320a upregulates LRWD1 through AGO2-FXR1 complex switching

It has been found that the dysregulation of miR-320a in osteosarcoma and soft tissue sarcomas interferes with the progression of tumor development [25–28]. miR-320a was first reported in 2014 to be highly associated with the development of leiomyosarcoma (LMS) and undifferentiated pleomorphic sarcoma (UPS), and the expression of potentially involved genes, including IMP3, ROR2, MDM2, CDK4, and UPA, was shown to be correlated with chromosomal modification and tumor development/progression of LMS and UPS [28]. A study in 2019 identified miR-320a as a tumor suppressor by showing that miR-320a overexpression reversed nicotinamide nucleotide transhydrogenase antisense RNA 1 (NNT-AS1)-induced proliferation, survival and mobility of the osteosarcoma cell line U2OS and that a miR-320 inhibitor partially rescued these functions of OS-732 cells [26]. However, our findings in the current study suggest that miR-320a acted as an onco-miR in promoting NT2D1 cell proliferation and that the miR-320a inhibitor suppressed proliferation. However, the evidence showed that miR-320a acted as a tumor suppressor miRNA by inhibiting NT2D1 cell migration and invasion. We suspect that this abnormal phenomenon may be due to the interaction between miR-320a and LRWD1, a unique regulatory factor in testicular cancer cells.

There is already much literature supporting the idea that miRNAs promote the translation of proteins involved in cancer and suggesting that they can regulate gene expression in more ways than previously thought. AGO2, which has been reported to have a function related to many miRNAs, including miR-122, miR-132 and miR-302 [19, 29, 30], is one of the core components of the miRNA-induced silencing complex (miRISC) [20, 31]. However, it has been frequently reported that in G0 cells and senescent cells, low expression of GW182 and high expression of FXR1 and AGO2 lead to upregulation of gene expression [32–34]. In fact, early in 2007, Vasudevan et al. have reported that the human miRNA miR-369-3 directs the association of cell cycle arrest proteins and TNFα upregulation with AREs to activate translation [34]. The authors provided strong evidence regarding the association of AGO2 and FXR1 binding to AREs throughout the process of translation activation and switching miRNA-initiated gene repression to activation [12]. Subsequently, studies regarding miRNA-mediated gene translation activation further confirmed and clarified this rare phenomenon [35, 36]. Interestingly, in addition to playing roles in senescent cells, the findings of Corey et. al imply that miRNAs participate in more complex regulation of protein expression. In some circumstances, such as in oocytes [36] and mitochondria [37], miRNAs help promote mRNA expression while mRNAs lack the 5′-cap and targeting TOP mRNAs (5′TOP), a structural trademark core of translational regulatory elements. Jame-Chenarboo et al. also reported that AGO2 and FXR1 are necessary for this process [38] and provided evidence that are similar to ours. AGO2 and FXR1 provide an explanation for the promoting effect of miR-320a on LRWD1 expression and the subsequent cell behavior alterations. Besides, AGO2 was reported to bind with eIF4E to forms a “closed loop” even in the absence of poly(A) β and related proteins and directly activates translation to increase gene expression [39]. In contrast, miRNA-320a inhibitor reduced the gene expression of eLF4E and eEF2, indicating that miRNA-320a promotes gene transcription through these regulators. eIF4E is involved in ribosome biogenesis and is thought to not only stimulate ribosomal protein mRNA translation *in vivo* and in vitro, but also increase transcriptional activation [40]. However, most of the details remain unclear and remain further experiments. Nevertheless, our results still showed the importance of miR-320a in increasing LRWD1 expression by through regulating AGO2/FXR1 complex, eLF4E, and eEF2 expression in the LRWD1 3′-UTR. The current study reveals a new phenomenon in which miR-320a promotes LRWD1 expression in NT2D1 cells to affect cell behaviors, and the downstream effect is worthy of further investigation.

### The role of miR-320a and LRWD1 in cellular antioxidation

In 2012, we confirmed the presence of nuclear transcription factor NF-κB binding sites on the LRWD1 promoter through chromatin immunoprecipitation (ChIP) and gel electrophoresis mobility change detection (EMSA) analysis methods, revealing that NF-κB positively regulates the activity of the LRWD1 promoter [4]. NF-κB can regulate proliferation, promote abnormal growth and anti-apoptosis of cancer cells, enhance cell inflammatory factors, and modulate the level of oxidative stress [41, 42].

Our results are consistent with previous results. When cells were treated with ROS, such as H2O2 and nitric NO donor SNPs, the expression of LRWD1 was greatly enhanced. Surprisingly, oxidative stress also enhanced the expression of miR-320a. We speculate that external factor-induced oxidative stress may cause upregulation of miR-320a, thereby further enhancing it and regulating downstream oxidative factors. A previous study showed that manipulating the expression of miR-320a could regulate the induction of oxidative stress. Changing the level of miR-320a regulated the expression of PFKm through the 3′UTR and influenced glycolysis, thereby affecting the Warburg effect in cancer cells [43]. In that case, the regulation of miR-320a/LRWD1 was also involved in oxidative adaptation, so the potential participation of miR-320a/LRWD1 in the Warburg effect of testicular cancer cells is interesting and is worthy of investigation. Furthermore, miR-320a was reported to be overexpressed in osteoporotic bone tissues in 2018 [44]. Laura De Ugart et al. reported that miR-320a regulated osteoblast genes and was involved in oxidative stress. Overexpression of miR-320a increased cell proliferation and oxidative stress levels and decreased the mineralization capacity of osteoblasts, which is consistent with our findings that miR-320a and LRWD1 levels were also increased in response to external oxidative stress in NT2D1 cells.

We further discovered in 2018 that another important transcription factor that regulates antioxidant enzymes, NRF2, is also involved in the regulation of LRWD1 expression and the response to oxidative stress [45]. Therefore, when we confirmed that miR-320a is an important regulatory miRNA of LRWD1, we also wanted to determine the mutual regulation of miR-320a and NRF2 under oxidative stress conditions. The results confirmed that miR-320a is the upstream gene of NRF2. When cellular oxidative stress is increased, by regulating NRF2 and KEAP1, cancer cells can increase the expression and activity of NRF2 or reduce the binding activity of KEAP1 to stabilize NRF2 and activate downstream antioxidant mechanisms, thereby escaping the toxic effects of anticancer drugs and contributing to the development of cancer. An increase in NRF2 content increases the expression of heme oxygenase-1 (HO-1) and BTB domain and CNC homology 1 (BACH1) and the related factors associated with cancer tumor invasion and metastasis [46, 47]. In our case, the expression of miR-320a, LRWD1 and NRF2 was increased, which is conducive to the survival of testicular cancer cells. From this perspective, targeting miR-320a or LRWD1 seems to be a promising antitesticular cancer treatment strategy.

**Figure 8:**
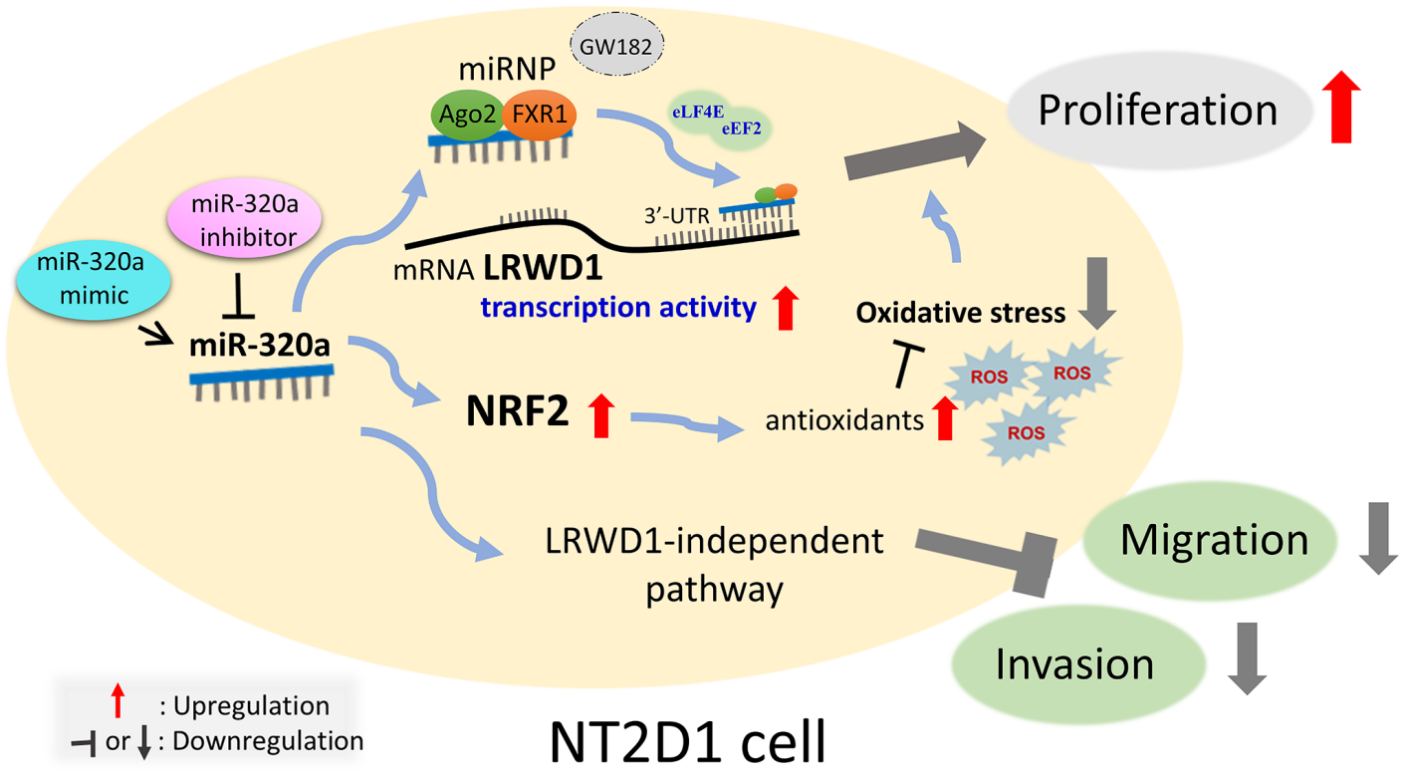
**Schematic diagram of miR-320 targeting human LRWD1 mRNA and influencing biological functions.** miR-320a exerts a favorable regulatory effect on the expression of LRWD1 and NRF2 genes and proteins in response to oxidative stress. miR-320a potentially has a significant impact on cell behavior via interacting with the miRNP AGO2/FXR1 complex, eLF4E, and eEF2. These interactions may facilitate binding to LRWD1 and enhance mRNA transcriptional activity. Moreover, there is a positive correlation between miR-320a and LRWD1 and the proliferation of NT2D1 cells. However, they have a detrimental effect on cell migration and invasion. Therefore, under conditions of oxidative stress, elevated levels of miR-320a and LRWD1 promote the proliferation of NT2D1 testicular cancer cells.

In summary, as shown in Figure 8, the current study reveals for the first time that miR-320a positively regulates the expression of LRWD1 and NRF2 genes and proteins and is involved in oxidative stress. miR-320a may play a key role in cell behavior through the miRNP AGO2/FXR1 complex. Interestingly, miR-320a and LRWD1 are positively correlated with the proliferation of NT2D1 cells but negatively regulate cell migration and invasion. The results indicate that miR-320a and LRWD1 are increased in the face of oxidative stress, which is conducive to the growth of NT2D1 testicular cancer cells.

## MATERIALS AND METHODS

### Cell culture

NTERA-2cl. D1 (NT2D1) human testicular cancer cells were purchased from the Food Industry Research Institute of Taiwan. Dulbecco’s modified Eagle medium (high glucose powder, H-DMEM) was supplemented with 5% fetal bovine serum (FBS), 5% calf serum (BCS), 1% antibiotic-antimycotic solution, and 1% L-glutamine solution according to the cell culture instructions. Cells (3.2 × 10^6^ cells/ml) were placed in a total of 3 ml in a 6-cm dish and incubated in a 37°C, 5% CO_2_ cell incubator.

### Lentiviral-based plasmid transfection

NT2D1 cells at 50% confluence were used for lentivirus transfection. A mixture of 100 μl of lentivirus and 1 ml of H-DMEM (supplemented with 5% FBS, 5% BCS, 1% antibiotic-antifungal solution, and 1% L-glutamine solution) was added to NT2D1 cells in a 6-well plate, and the cells were incubated in a 37°C, 5% CO_2_ cell incubator for 24 hours. After 24 hours of infection, medium containing 25 mg/ml puromycin was added for 24 hours of cell selection. Cells with resistance to puromycin were amplified and cultured, and the knockdown efficiency was confirmed by western blotting and real-time qRT-PCR analysis.

### LRWD1 expression measurement

NT2D1 cells (5 × 10^3^/ml) were seeded into 96-well plates, and pMIR-REPORT, pMIR-LRWD1 3′UTR, and pMIR-LRWD1-3′UTRΔmiR-320a were transfected into NT2D1 cells. To use Lipofectamine 3000 reagent for cell transfection, two transfection reagents were first prepared, namely, P3000TM Enhancer reagent and Lipofectamine 3000 reagent, and then pMIR-REPORT (300 ng), pMIR-LRWD1 3′UTR (300 ng), and pMIR-LRWD1-3′UTRΔmiR-320a (300 ng) were added separately into labeled mixing tubes at room temperature for 10–15 minutes. Then, the transfection reagent mixture was added to the 96-well plate for 24 hours. After the transfection medium was removed, 20 μl of 1× passive lysis buffer (PLB) was added for 15 minutes and mixed by rotation for 10 seconds. Twenty microliters of cell lysate were transferred to a new 96-well plate protected from light, and then 50 μl of Dual-Glo luciferin-luciferase reagent (LAR II) was added to measure firefly luciferase activity. Fifty microliters of Stop and Glo reagents were added to measure the luciferase activity of Renilla.

### RNA extraction and qRT-PCR

After extracting RNA, reverse transcription to generate cDNA was performed by using a Magic RT Master Mix cDNA synthesis kit. For reverse transcription, the following reaction conditions were used: 2 μg RNA, 10 μl 2× Magic RT Master Mix, 1.5 μl 10× reverse transcriptase buffer, 1 μl MultiScribe reverse transcriptase, 4.16 μl nuclease-free water, and 3 μl 5× hsa-miR-320a RT primer. A TT100™ thermal cycler (Bio-Rad, USA) was used for RT-PCR under the following conditions: 42°C for 30 minutes and 85°C for 5 minutes. After the reaction, the cDNA was stored at 4°C.

One microliter of cDNA was mixed with primers, DEPC-Water and 2× IQ2 SYBR Green Fast qRT-PCR system master mix, and then target gene-specific primers were added for real-time qRT-PCR analysis.

### Western blot analysis

For this assay, an 8% SDS-PAGE gel was placed in 1× running buffer, loaded with the denatured protein samples, and run at 60 V and 20 mA for 60 minutes for the stacked gel and 100 V and 20 mA for 120 minutes for the separation gel. After the electrophoresis run was finished, membrane transfer was performed in 1× transfer buffer and a semidry transfer unit at 20 V and 300 mA for 30 minutes. After membrane transfer, the membrane was blocked with blocking buffer at room temperature for 1 hour. The primary antibody (1:3000-1:5000) was incubated overnight (16–18 hours) at 4°C. Then, the PVDF membrane was washed with 0.1% TBST buffer 5 times for 10 minutes, and an HRP-conjugated secondary antibody (1:10000) was added for 2 hours. The membrane was reacted with enhanced chemiluminescence (HRP substrate luminal reagent: HRP substrate peroxide solution = 1:1) for 10 minutes away from light and allowed to develop color to measure the expression of the target protein.

### WST-1 cell viability/proliferation assay

NT2D1 cells (5 × 10^3^ cells/ml) transfected with shLuc, shmiR-320a, miR-320a mimics, or miR-320a inhibitor were inoculated into 96-well plates and cultured for 24 hours. WST-1 reagent was added to H-DMEM at a ratio of 1:10, and 100 μl/well aliquots were placed in the dark for 2 hours. A Synergy HTX multimode microplate reader was used to measure the absorbance at 440 nm and to determine the cell content.

### Wound-healing assay

We used the wound-healing test to investigate NT2D1 cell migration. We observed the cells at three time points: 0 hours, 12 hours and 24 hours. After transfection of the cells with shLuc and shLRWD1, the effects of additional miR-320a mimic and miR-320 inhibitor treatment on cell migration were investigated. Untreated cells were used as controls. After the cells were imaged, ImageJ was used to analyze the proportion of cells in the central area and the ability of the cells to migrate after gene manipulation.

### Cell invasion assay

NT2D1 cells transfected with shLuc and shLRWD1 were seeded in 4-well plates at 1 × 10^5^ cells/ml and incubated for 24 hours before transfection with the miR-320a mimic and miR-320a inhibitor. Matrigel (0.1%) was mixed with H-DMEM, and 100 μl/Transwell was added and placed in a 37°C, 5% CO_2_ cell incubator for 2 hours. Transfected NT2D1 cells were seeded into Matrigel-coated Transwell inserts, and the bottom chambers were filled with 650 μl H-DMEM and incubated for 24 hours for the cell invasion assay. After 24 hours, the 24-well plate was removed for Transwell washing. After removal of the Transwell, 1 ml of 3.7% formaldehyde was added at room temperature for 10 minutes to fix the cells. Next, 1 ml of 99.9% methanol was added, and the cells were placed at room temperature for 20 minutes. Then, 1 ml of 1% crystal violet was added, and the cells were stained for 15 minutes at room temperature away from light. After removal of the staining solution, images were taken using a microscope, and ImageJ was used for cell counting and statistical analysis.

### Statistics

All data are presented as the mean ± standard error of the mean (SEM) of at least three independent experiments. One-way analysis of variance was used to establish differences among groups using SPSS 22.0 (SPSS Inc., USA), and multiple comparisons with significant differences were further verified by using Tukey’s post hoc test (Studentized range distribution) to avoid type I errors. Differences with *p* < 0.05 were determined to be statistically significant.
